# Thorough documentation of the accidental aspiration and ingestion of foreign objects during dental procedure is necessary: review and analysis of 617 cases

**DOI:** 10.1186/s13005-016-0120-2

**Published:** 2016-07-22

**Authors:** Rui Hou, Hongzhi Zhou, Kaijin Hu, Yuxiang Ding, Xia Yang, Guangjie Xu, Peng Xue, Chun Shan, Sen Jia, Yuanyuan Ma

**Affiliations:** Department of Oral Surgery, State Key Laboratory of Military Stomatology & National Clinical Research Center for Oral Diseases & Shaanxi Clinical Research Center for Oral Diseases, School of Stomatology, The Fourth Military Medical University, Xi’an City, Shaanxi Province 710032 China; Department of Stomatology, Research Institute of Surgery & Daping Hospital, The Third Military Medical University, Chongqing City, 400042 China

**Keywords:** Aspiration, Ingestion, Foreign objects, Dental procedure, Documentation

## Abstract

**Objectives:**

To review the cases of accidental aspiration and ingestion of foreign objects during dental procedure, and to emphasize the importance of thorough documentation of the accidents.

**Methods:**

A comprehensive search on (dental procedure/treatment/practice), (aspiration/inhalation), and (ingestion/swallow) was performed for all years before 1st October 2014 available. The statistic analysis was made on the variables including journals and reported year, patients’ age, gender, general conditions, dental procedure and location for procedure, foreign objects, site of involvement, possible causes, anesthesia during procedure and treatment, symptoms, treatment time and treatment modality, follow-up, and so on.

**Results:**

A total of 617 cases reported by 45 articles from 37 kinds of journals were included and analyzed. Most reports made detailed record. While some important variables were recorded incompletely, including patient’s general conditions, location for procedure, clinical experience of the involving dentists, tooth position of procedure, possible causes, and anesthesia during procedure and treatment for the accident.

**Conclusions:**

Aspiration and ingestion of foreign objects are rare and risky complication during dental procedure. Each accident should have thorough documentation so as to provide enough information for the treatment and prevention.

## Background

Aspiration and ingestion of foreign objects are potential complications that can occur during dental procedure, such as root canal therapy, implantation, extraction, and even routine examination. The foreign object included endodontic instruments, implant components, burs, posts, teeth, orthodontic brackets, restorations and even dental mirror and irrigation needle [1–5].

The incidences of aspiration and ingestion in dental procedure have been reported by many articles and reviews. As early as 1971, Grossman [6] determined that 87 % of foreign bodies entered the alimentary tract, whereas 13 % aspirated into the respiratory tract. Susini G et al. [7] reported that the incidences of aspiration and ingestion in root canal treatment were 0.001 per 100 000 and 0.12 per 100 000, respectively. From different dental college hospitals in Japan, the ingestion of foreign objects was reported 0.0041 and 0.0044 % [8, 9]. Moreover, the occurrence (cases/dentists) per year was 0.018, which was very close to the figure of 0.021 reported from two French insurance companies representing 24,651 French general dental practitioners over an 11-year period [7].

The literature also showed that although 90 % of ingested foreign objects could pass through the gastrointestinal tract uneventfully, there are roughly 10 % require endoscopic removal, while still 1 % will ever require operation [6, 8, 10, 11]. Although bronchoscopy has been reported 99 % effective on retrieve the aspirated foreign objects, the complication rate is between 2.4 and 5 % [12].

Many factors are reported related to the aspiration and ingestion. For example, patients’ medical and mental condition, use of local anesthesia or intravenous sedation, difficulty of access, compromised direct view, and so on [2–5]. However, these factors are still in controversy. There were also some important variables recorded incompletely from the literature, such as tooth position of procedure, clinical experience of the involving dentists, and anesthesia during procedure. In addition, many articles even did not report the necessary information of the cases. Moreover, there were hardly any review on making comprehensive record and discussion of accidental aspirated and ingested cases.

Therefore, it has necessity to strengthen the thorough documentation so as to arouse the dental personnel’s attention, and further to facilitate analysis of the reasons, accumulation of the experience and lessons, and summary of the prevention and treatment measures on accidental aspiration and ingestion.

## Methods

### Literature search

An extensive literature search was conducted in four electronic databases: PubMed, Cochrane Library, ScienceDirect, and Embase databases. The following filters were used in the search strategy: date (1970/01/01 to 2014/10/01) and species (humans) filters in PubMed, and only date (1970–2014) filter for the remaining three databases. The reference lists of all relevant articles were also screened manually to identify further potentially relevant articles.

The inclusion criteria were as follows: (1) case reports, case series, review articles and retrospective studies; (2) studies reporting the accidental aspiration and ingestion of foreign objects during the dental procedure, dental treatment, and dental practice. (3) studies reporting at least the following information: dental procedure and foreign objects, site of involvement and symptoms, treatment modality and follow-up. (4) studies published in English.

The exclusion criteria were as follows: (1) studies contained limited data including conference abstracts and letters to journal editors, and opinion articles; (2) studies reporting the accidents happening in time other than dental treatment; (3) studies only reporting the prevention and treatment measure of aspiration or ingestion without cases.

Two reviewers independently judged the study eligibility, and any disagreement was resolved by consensus.

The descriptive variables were extracted and collected thoroughly, including journals and reported year, patients’ age, gender, general conditions, dental procedure, location for procedure, clinical experience of the involving dentists, tooth position of procedure, possible causes, foreign objects, site of involvement, symptoms, treatment time and treatment modality, anesthesia during procedure and treatment for the accident, and follow-up.

### Statistical analysis

SPSS version 13.0 for Windows was used for statistical analysis. The descriptive statistics were made on all the descriptive variables from the selected articles.

## Results

A total of 617 cases reported were included and analyzed in this review. Most cases were recorded in detail, while some important variables were incomplete. The statistical analysis results were listed below based on the different descriptive variables.

### Variables recorded in detail

#### Journals and reported year

**Fig. 1:**
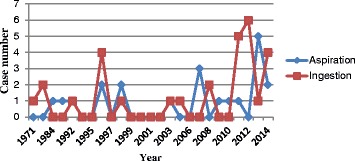
Analysis on cases number and its happened year. Showed cases number and their reported year (except four reviews)

There were altogether 45 articles published by 37 kinds of journals on aspiration and ingestion during dental procedure. Table 1 showed the analysis on cases number from the articles. Figure 1 showed cases number and their reported year (except four reviews).

**Table 1:** Analysis on cases number from the articles (case number)

	Year	Aspiration	Ingestion	Total
Review from France [7]	1994–2004	44	464	508
Review from Japan [8]	2008–2009	0	11	11
Review from Japan [9]	2006–2010	0	23	23
Review from USA [13]	1992–2002	1	25	26
Case reports	1971–2014	20	29	49
Total number		65	552	617

More than 80 % of the articles (37/45) were from dental journals. Among them, 19 articles were from comprehensive dentistry [8, 9, 11, 13–28], 6 from oral sugery [10, 29–33], 6 from endodontics [7, 34–37], 3 from prosthodontics [1, 38, 39], 2 from implantation [4, 40] and 2 from orthodontics [41, 42]. The others 8 were from the fields of gastroenterology [43], respiration [44], laryngology [45], pediatrics [46] and comprehensive medicine [4, 47–49].

#### Age and gender

**Fig. 2:**
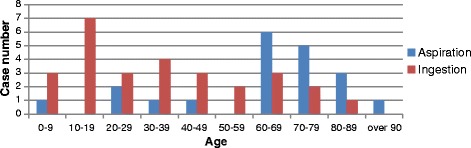
Analysis on the patient’s age of the cases. Showed that aspiration and ingestion were more seen in patients at 60–79 years old and 10–19 years old, respectively

Figure 2 showed aspiration and ingestion were more seen in patients at 60–79 years old and 10–19 years old, respectively. Of all the 49 cases in case reports, the aspiration and ingestion case number were 18 and 15 in male, 2 and 12 in female. There were even 2 cases did not specify gender [27].

#### Dental procedure and foreign objects

**Fig. 3:**
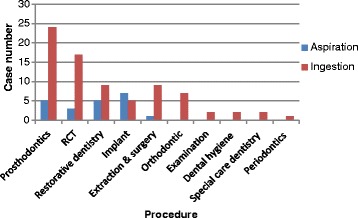
Analysis on the dental procedure of the cases. Showed that aspiration happened more during implantation, prosthodontics, and restorative dentistry. Ingestion happened more during prosthodontics and RCT

Aspiration happened more during implantation [10, 19, 30, 33, 40, 46], prosthodontics [13, 14, 20, 28], and restorative dentistry [11, 14, 22, 28, 45]. Ingestion happened more during prosthodontics [9, 13, 29, 39] and RCT [9, 11, 13, 16–19, 24, 27, 34, 35, 37, 43, 49] (Fig. 3). Table 2 listed the top five kinds of foreign objects that were aspirated and ingested in the case reports and reviews.

**Table 2:** The top five kinds of foreign objects aspirated and ingested (case number)

Foreign objects	Case reports		Foreign objects	Reviews	
	Aspiration	Ingestion		Aspiration	Ingestion
Endodontic file (& reamer)	3	11	Prosthesis & crown	32	171
Screwdriver (& screw)	6	6	Bur	0	126
Crown & bridge	3	1	Endodontic file	1	57
Bur & drill	3	0	Inlay core	7	49
Rubber dam clamp	0	2	Broach	0	27

#### Site of involvement and symptoms

For the aspirated cases, 7 cases were found foreign objects at right bronchus [10, 11, 14, 22, 30, 31, 33], 6 at left bronchus [15, 19, 36, 44, 45, 48], 5 at right lung [13, 14, 20, 28, 40], 1 at lung without description on left or right [28], and the other one at the piriform fossa [10]. For the ingested cases, 24 cases were found at stomach [4, 9, 11, 16, 18, 21, 27, 29, 32, 35, 37, 42, 47, 49], 10 at intestine [9, 27, 46], 11 at stomach and intestine [8], and esophagus (5 cases) [9, 23, 25].

Of all the 49 cases, 9 aspirated cases had symptom, including 7 cough [10, 15, 19, 22, 31, 36, 40], 1 pain [20], 1 gag [28]. Four ingested cases had symptoms, including 3 pain [11, 43, 46] and 1 cough [34]. All the reviews had no description on the symptoms.

#### Treatment time, modality and follow-up

Table 3 showed two thirds of the aspirated cases (13/20) got immediate treatment [10, 11, 14, 15, 19, 22, 30, 31, 33, 40, 44, 45, 48], while nearly 40 % of ingested cases (12/29) got observation with foreign objects excreted 2 days to 2 weeks later [18, 21, 24, 27, 29, 34, 35, 37, 39, 41, 49]. Among the 34 ingested cases in reviews from Japan [8, 9], only 3 cases retrieved by endoscopic procedure immediately, the others passed through the gastrointestinal tract in a 10-day period.

**Table 3:** Treatment time of the aspirated and ingested cases (number)

	Immediate	2d	3d	4d	5d	7d	2w	5w	2 m	1y	3y	7y	Total
Aspiration	13	2	1	1				1		1	1		20
Ingestion (treatment)	7	1	1		1	3	2		1			1	17
Ingestion (observation and excretion)		2	5	1	2	1	1						12

Of all the 20 aspirated cases, 15 cases had the foreign objects successfully retrieved by bronchoscopy (7 flexible [10, 14, 28, 40, 44, 45], 5 rigid [22, 30, 31, 33, 48] and 3 without description [15, 19, 36]) and 1 case by laryngoscopy [10]. Three cases failed to retrieve the object after bronchoscopy, including 1 observed with excretion until 6 months later [14], 1 had lung wedge resection 3 days later [14], and 1 got recall every month but without final result reported [36]. The last one case got the lobectomy of right lobe when the dental impressions were found aspirated 1 year later [20].

Of all the 29 ingested cases, 12 got foreign bodies excretion, including 10 with observation before [21, 24, 27, 29, 34, 35, 39, 41, 49] and 2 with immediate endoscopy failed before [18, 37], 15 had the objects retrieved by endoscopy (7 immediate [11, 17, 23, 25, 32, 34, 42], 8 several-day later [1, 4, 11, 16, 29, 43, 47]), and the other 2 had laparatomy [38] and colostomy [46], respectively.

For follow-up, only one case reported happening acute airway obstruction after bronchoscopy [33]. The symptom finally disappeared after suitable treatment. There were no adverse events or description to the other cases.

### Variables recorded incompletely

#### General conditions

There were only 12 cases (12/617) reporting patients with general disease, including 6 cases of cerebrovascular disease [9, 19], 2 cases of tumor excision [33, 38], 1 case of attention deficit hyperactivity disorder [15], 1 case of low intelligence quotient [17], 1 case of dental retardation [21] and 1 case of cleft palate [41].

#### Location for procedure and clinical experience of the involving dentists

In the case reports, there were 14 cases happened at private clinic/hospital [10, 15, 16, 19, 22, 25, 30, 34, 36, 37, 40, 41], 17 cases happened at dental clinic or department in hospital or college hospital [1, 4, 14, 17, 18, 21, 23, 24, 27, 32, 33, 35, 38, 39, 42, 49]; the other 18 cases had no description. In four reviews, there were 60 cases from dental clinics of university hospital [8, 9, 13], the other 508 cases were from general dental clinic [7].

Only two reviews from Japan made detailed analysis on clinical experience of the involving dentists [8, 9]. Both of them thought the accidental ingestion occurred more frequently when procedures were being conducted by practitioners with less experience (5 to 10 years [8], less than 5 years [9]).

#### Tooth position of procedure and causes

In the case reports, only 16 cases [10, 15–17, 23, 24, 27, 28, 31, 34, 35, 49] were recorded detailed tooth position of procedure. Two reviews reported the ingestion occurred more frequently during treatment of lower molars [8, 9]. The other two reviews had no description [7, 13].

In the case reports, only 14 cases mentioned the causes, including 6 cases of closing mouth and moving head [15, 17, 25, 34, 41, 46], 3 of discomfort and uncooperative [18, 23, 33], 2 of gagging reflex [24, 31], 2 of instrument fatigue [21, 27], and 1 of no floss tie [11]. All the reviews had no description on the causes.

#### Anesthesia during procedure and treatment for the accident

During the procedure, only 5 cases received local anesthesia (3 aspirated [1, 23, 43], 2 ingested [31, 33]), 2 cases received sedation (1 aspirated [10], 1 ingested [32]). The other 43 cases and 4 reviews did not receive any anesthesia nor had the record.

During the treatment for aspiration, there were 10 cases received general anesthesia [14, 15, 19, 20, 30, 31, 33, 36, 48], 3 cases received sedation [10, 40], 2 cases received local anesthesia [22, 44], and the other 5 cases had no record [11, 14, 28, 45]. During the treatment for ingestion, there were 7 cases received general anesthesia [17, 23, 25, 29, 39, 46, 47], 2 cases received sedation [32, 42], the other 20 cases did not receive any anesthesia nor had the record.

## Discussion

Aspiration or ingestion of foreign objects including instruments, materials or even tooth is a relatively uncommon risk during dental procedures [14]. Yet, it is reported to be the second most common reason for foreign body aspiration in the lung [13]. Actually, the accidents could happen during various dental procedures due to some factors and associated with certain incidence, suggesting the importance of patient’s safety and instituting precautions and countermeasures at all times.

However, most literature only reported one or several cases with limited information on the description of the accidents. There were hardly any review on making comprehensive record and discussion of accidental cases.

Therefore, thorough documentation of the accident is stressed in this study so as to arouse the dental personnel’s attention, and further to facilitate analysis of the literature, and summarize the prevention and treatment measures on accidental aspiration and ingestion.

In this article, a total of 617 cases reported by 45 articles were reviewed. The statistical analysis was based on the different descriptive variables. Most reports made detailed records on patients’ age, gender, dental procedure and foreign objects, site of involvement and symptoms, treatment time and modality, and follow-up.

Figures and tables showed there were more accidental cases happened in recent years. Aspirated and ingested cases were more seen in older patients and younger patients, respectively. Male patients suffered more cases than female patients. And the cases were more seen in the fine, cumbersome, and time-consuming procedure. In addition, any kinds of foreign objects could be aspirated or ingested regardless of the shape, size, and even length.

In aspirated cases, foreign objects were found more at right bronchus or lung because the connection from the trachea to the right bronchus is a less marked angle; moreover, the right bronchus has a greater diameter than the left [4, 5, 50]. In ingestion cases, the site of involvement was probably related with the time after the accident. If the checking time is short after the ingestion, the object may be in the stomach; otherwise it will be in the intestine. Since half of the aspirated patients and more than 90 % of the ingested patients had no symptom, it suggested to us that once the instruments, material and even tooth could not be found during procedure, possible aspiration and ingestion might be detected.

From the results, it suggests that once aspiration is confirmed, immediate treatment should be done, since the majority of the cases need endoscopy or even surgery. However, once ingestion is confirmed, observation could be performed until the foreign object excreted. If there is no possible of excretion [18], endoscopy should be chosen. The follow-up also suggested that the treatment was suitable and the complications were under control. The prognosis was pretty good.

However, there were still some important variables recorded incompletely. It showed that only 12 cases reported patient’s general conditions. The finding contradicts the widely held belief that patients with a neuromuscular disease or a physical handicap are at high risk of aspirating or ingesting dental foreign objects.

The results also showed that even 18 cases did not report the location of the accident, that only a small number of cases recorded detailed tooth position, and that only a few cases recorded the clinical experience and occupations of the involving dentists though it was found even lecturer and assistant professor with more than 20 years’ experience can make mistakes in this respect [9].

As for the causes, it has been reported [4, 5, 14, 51] that psychotic individuals, alcoholics, mentally disabled individuals, patients who are nervous or restless, and patients who wear dentures ascribed to reduced tactile sensitivity of the palatal mucosa are at high risk of inhaling and swallowing foreign objects, but there were only few records in the dental procedure literature we studied [15, 21]. On the contrary, other possible factors were not recorded in detail, including supine positioning, excessive gag reflex or unexpected patient movement, inadequate lighting, ineffective assistants, instrument fatigue, difficulty of access (posterior areas), and compromised direct view.

In addition, local anesthesia and intravenous sedation had been suggested as the possible reason, since sedation decreases the protective swallowing and coughing reflexes [14, 15]. However, the reason was in controversy since most cases during the procedure in the study did not receive any anesthesia nor had the record. The anesthesia during the treatment for the accidents was recorded incompletely, too.

From above, it could be seen that the missing information have had an effect on the comprehensive analysis of the results. Therefore, it is necessary to emphasize the importance of the thorough documentation and make each dental personnel to do a comprehensive understanding, learning and mastering the treatment and prevention on the accidental aspiration and ingestion.

On the one hand, the proposed treatment algorithm is critical for the management of the complications which could be summarized from the literatures.

Firstly, when accidental event occurs, it is essential that clinicians and their staff remain calm and composed. The patient must be reassured and carefully evaluated [15, 16, 52].

Secondly, thorough clinical and radiological evaluations are required [16]. Early location of an aspirated or ingested foreign body facilitates appropriate and timely treatment management and referral [37]. In cases of aspiration, both posterior-anterior and lateral X-ray films should be taken to confirm the location of foreign objects in the respiratory tract [19]. If the objects (e.g. impression materials or resins) are made of substances that lack of radiopacity, diagnostic bronchoscopy or computed tomography is necessary for their localization.

Thirdly, a prompt decision must be made whether to actively remove the object or to let it pass naturally. The subsequently appropriate actions must be taken to prevent potentially serious complications, and may ultimately save the patient’s live [15, 16].

When the object is located in the oral cavity, finger sweeps is the simplest way. When the object is impacted in the airway, noninvasive procedures for managing airway obstruction include back blows in infants, the Heimlich maneuver, abdominal or chest thrusts in pregnant or obese patients [53].

Once aspirated object is confirmed, urgent management with a flexible or hard fiber optic bronchoscope should be performed [54, 55], otherwise it can obstruct the airway [41] or cause pneumonia or a pulmonary abscess [11]. This technique has a success rate of 99 %, with a failure rate of 2.4 to 5 % [10]. And the failed cases require surgical intervention of lobectomy.

If an object is swallowed and impacted in the esophagus, prompt removal is required because the esophagus lies in close proximity with the thoracic great vessels, the pericardium, the pleura and the tracheo-bronchial passages [56–58]. If the object goes into the stomach, there is a greater than 90 % chance, especially for some small (less than 2 cm), blunt objects, that it will pass through the gastrointestinal tract as a result of peristaltic movement without complications [30, 57–59]. Conservative management should include radiographic surveillance and periodic stool inspection [52]. However, sharp, pointed objects are associated with a higher risk of perforation. The perforation is most likely to take place in the esophagus, the pylorus, the duodenum, the duodenojejunal flexure and the ileocaecal region [60]. Thus, early endoscopic removal should be undertaken [55]. If patients develop symptoms of pain, nausea, vomiting, tenderness or abdominal guarding, perforation should be suspected, and if objects remain lodged longer than 2 weeks, surgical intervention is required [17, 59].

Fourthly, the patient should be observed until the object is removed or expelled [21]. A post-operative radiograph should be taken to confirm that the aspirated or ingested instrument has been excreted or removed [52].

Fifthly, thorough documentation of the accident is required as discussed above. Further documentation may include notation of initial and follow-up medical care, clinical experience of the involving assistant or nurse, copies of radiographic reports confirming the diagnosis and notation of removal/expulsion of the objects [21].

On the other hand, prevention through precautionary methods is the most appropriate method to minimize the occurrences of aspiration of dental instruments.

Firstly, every dental personnel should consider the possibility of such emergencies in its standard operating procedures and be well prepared for them [42]. One must be educated and trained regularly to recognize emergencies and how to prevent and minimize adverse events in the work environment [61, 62]. Individual responsibilities must be delegated to offset any confusion in the event of an emergency so as to organize smooth support and cooperative procedures that can be implemented promptly if accidental ingestion or aspiration occurs [9]. To have available the name, address, and telephone number of an endoscopist and a hospital where full service is available is also necessary.

Secondly, patient’s thorough medical and dental history should be reviewed [50]. Special considerations should be associated with those patient populations at high risk, and schedule short appointments to them during the morning are most effective [19].

Thirdly, patients should have enough pre-operation educattion. The dental stuff must ensure complete cooperation and active involvement of patients and their accompany [61].

Fourthly, all the instruments should be periodically check and carefully examined before use for signs of wear or work fatigue and replace those that warrant replacement [10, 11, 21, 42]. For example, burs should be fully seated into the handpiece and locked into position [36]. Dental mirrors should be screwed in tightly before being inserted in the mouth [1]. Broken burs and instruments should be retrieved and matched up with retained fragments to ensure that all pieces have been recovered [10].

Finally, standard operating procedures with precautions must be taken during any practices. These precautions include appropriate anesthesia and treatment selection, proper body and head positioning, adequate lighting and four-handed dentistry with an attentive assistant and high-speed evacuation, routinely use of a rubber dam and a properly fitting clamp [10, 21], using a 4 × 4 inch gauze as a protective barrier in the oral cavity distal to the working area [1, 13, 15, 16], tethering small instrument, cast post, core and crown with a ligature to improve the gripping and reduce the possibility of falling from the hands [13, 63–67].

Of course, there were some limitations in the study. For example, articles in languages other than English were not included in the study. Articles published with only abstract or few details were also not included. These limitations may result in a slight bias in statistical analysis. In addition, some meaningful features were not analyzed. For example, the qualification of the dentists or doctors undergoing the procedure was not analyzed since they were recorded only in a few articles.

## Conclusions

Although aspiration and ingestion of foreign objects are rare and risky complication during dental procedure, thorough documentation of the accidental aspiration and ingestion of foreign objects during dental procedure is necessary so as to provide enough information for the treatment and prevention.

## Abbreviations

Not applicable
